# Face Blindness in Children and Current Interventions

**DOI:** 10.3390/bs13080676

**Published:** 2023-08-11

**Authors:** Weina Ma, Zeyu Xiao, Yannan Wu, Xiaoxian Zhang, Dongwen Zheng, Xue Lei, Chengyang Han

**Affiliations:** 1 Jing Hengyi School of Education, Hangzhou Normal University, Hangzhou 311121, China; 2 Zhejiang Philosophy and Social Science Laboratory for Research in Early Development and Childcare, Hangzhou Normal University, Hangzhou 311121, China; 3 School of Business Administration, Zhejiang University of Finance and Economics, Hangzhou 310018, China

**Keywords:** children with prosopagnosia, face blindness, face memory, face perception, intervention

## Abstract

Children with prosopagnosia, also known as face blindness, struggle to recognize the faces of acquaintances, which can have a negative impact on their social interactions and overall functioning. This paper reviews existing research on interventions for children with prosopagnosia, including compensatory and remedial strategies, and provides a summary and comparison of their effectiveness. However, despite the availability of these interventions, their effectiveness remains limited and constrained by various factors. The lack of a widely accepted treatment for children with prosopagnosia emphasizes the need for further research to improve intervention strategies. Last, three future research directions were proposed to improve interventions for prosopagnosia, including ecological approaches, the social challenges faced by children, and new potential intervention methods.

## 1. Introduction

Face recognition plays a crucial role in social interactions as it enables individuals to process various social categories of information conveyed by faces, including gender, age, and race. It allows for the quick identification and understanding of others [1]. Moreover, subtle facial cues can be utilized to discern trustworthiness and personality traits [2,3]. The ability to recognize familiar faces is fundamental to human cognition and vital for establishing and maintaining social connections. 

However, for individuals with prosopagnosia, this ability is significantly impaired. Prosopagnosia, also known as face blindness, is a condition in which individuals are unable to easily and accurately recognize others by their faces [4]. Prosopagnosia can manifest in two primary forms: acquired prosopagnosia (AP) and developmental prosopagnosia (DP). AP arises as a consequence of identifiable neurological damage, while DP, also known as congenital prosopagnosia, emerges without any accompanying intellectual deficits, emotional disturbances, object recognition difficulties, or acquired brain damage. Although cases of AP are relatively rare, DP is more prevalent in the general population, affecting approximately 2–2.5% of adults and 1.2–4% of children [5,6]. Research has revealed considerable comorbidity between prosopagnosia and other developmental and neurological conditions [7]. Prosopagnosia frequently co-occurs with autism spectrum disorder, Alzheimer’s disease, nonverbal learning disability, and epilepsy [8,9,10]. This implies that the high comorbidity with other developmental disorders must be considered during the diagnosis and treatment of prosopagnosia. The early identification of prosopagnosia symptoms and the concurrent treatment of co-occurring conditions would greatly aid in improving overall outcomes for children.

Notably, the study of prosopagnosia, particularly in the context of children, remains an area that calls for further research and exploration. While extensive research has been conducted on prosopagnosia in adults, there is a noticeable dearth of studies specifically focusing on the challenges faced by children with this condition [11]. This discrepancy highlights the need for further investigation into the unique challenges faced by children with prosopagnosia and the development of targeted interventions to support their social and cognitive development.

Prosopagnosia can have lasting effects on children’s ability to form and maintain social relationships. In one study, nearly all individuals with prosopagnosia had trouble recognizing people they knew well, such as friends, co-workers, and family members. This can cause them to feel embarrassed or belittled, and may increase anxiety and guilt about social interactions [12]. Furthermore, children with prosopagnosia may be at an increased risk of danger due to their inability to accurately recognize faces. For instance, they may mistake strangers for acquaintances or fail to recognize family members when outside the home, potentially leading to unsafe situations [13].

Given the significant impact of prosopagnosia on children’s social interactions and overall well-being, it is imperative to explore effective intervention methods to mitigate the challenges associated with this condition. By implementing targeted interventions, it may be possible to improve face recognition abilities in children with prosopagnosia and enhance their overall social functioning and quality of life.

A comprehensive search was performed in June 2022 and updated in May 2023 using Web of Science, PubMed, and Google Scholar. The search strategy combined the keywords “prosopagnosia” AND “face recognition, face processing, face memory, face perception, training, intervention”. Studies were excluded if participants had comorbid autism spectrum disorder or other developmental disorders in order to isolate interventions specifically targeting prosopagnosia. Given the limited evidence-based interventions for pediatric populations with prosopagnosia, no age restrictions were imposed in order to collect data across the lifespan. After applying these eligibility criteria, 38 articles were included in the final review. This process allowed us to thoroughly survey the current literature on prosopagnosia interventions and identify gaps needing further research, especially among children and adolescents.

## 2. Intervention Basis of Prosopagnosia

The mechanisms underlying face recognition during development differ from those involved in other forms of visual recognition. Specifically, defects in face-specific mechanisms can lead to prosopagnosia, a condition that arises when specific parts of the brain are damaged or lesions occur [14,15]. However, there is promising evidence suggesting that the face processing system exhibits neural reorganization capabilities following injury, indicating a degree of plasticity [16]. Moreover, research suggests that the face recognition system in children may possess greater plasticity compared to adults, highlighting the critical role of early intervention [17]. A study conducted by De Heering et al. utilized a digital version of the Benton Face Recognition Test (BFRT) to evaluate children and adults, revealing that children exhibited significantly longer correct response times than adults for both upright and inverted faces [18]. Additionally, there was an improvement in accuracy for upright faces between the ages of 6 and 12 years. Although some studies have proposed that face learning abilities may continue to improve into early adulthood, it is well established that recognition of inverted faces reaches its peak earlier in development [19]. Therefore, early intervention for children with prosopagnosia is crucial as their developing brains may be more responsive to targeted interventions aimed at improving face recognition abilities.

Face recognition is a complex cognitive process that involves multiple stages, including perceiving a face, encoding it into memory, and retrieving it later to determine familiarity and identity [20]. When any of these stages are impaired, the overall function of the face recognition system can be disrupted. Previous research has proposed three loci of impairment that can lead to prosopagnosia [16]. The first locus is the patient’s inability to accurately perceive faces. The second locus is the patient’s ability to correctly perceive faces but the inability to access stored face memories. The third locus involves the patient’s ability to perceive faces and make familiarity judgments, but this locus is unable to extract any semantic or identity information related to the person. In summary, the three loci refer to deficits in (1) face perception, (2) linking faces to identity/memory, and (3) deriving identity-specific semantic information from faces. Differentiating the locus of impairment can help better understand the specific deficits underlying prosopagnosia in a given patient.

Moreover, studies have suggested that AP can be divided into two subtypes: apperceptive prosopagnosia and associative prosopagnosia [21]. Patients with apperceptive prosopagnosia have difficulty perceiving faces and have damage to the fusiform gyrus, while those with associative prosopagnosia have difficulty remembering faces and have damage to the anterior temporal region. This classification is also reflected in patients with DP [15]. DP may manifest in multiple indistinct types. The researchers used four face tests to evaluate three DP patients, focusing on their abilities to perceive and remember faces. The tests included the Unknown Face Matching Test, Age Estimation Test, Familiarity Check Test, and Famous Face Recognition Test. The results exhibited significant variations among the three patients, suggesting that DP can be further divided into impaired face perception and impaired face memory types [22]. It is important to note that brain injury can also lead to prosopagnosia in children, and similar to adults, children may exhibit a separation between face perception and face memory [6]. In addition to the subtypes of prosopagnosia mentioned earlier, there are other factors that contribute to the complexity of this condition. For instance, individual differences in the severity and specific manifestations of prosopagnosia can widely vary [23]. 

Therefore, gaining a comprehensive understanding of the various types and subtypes of prosopagnosia, along with their specific impairments, is essential for the development of effective intervention strategies. By focusing on the specific areas of impairment and customizing interventions to meet the unique needs of children with prosopagnosia, it becomes possible to optimize their face recognition abilities and enhance their overall functioning in social interactions and daily life.

## 3. Intervention Strategies for Children with Prosopagnosia

Prosopagnosia can be improved through training [24], and the dissociation between face perception and face memory mechanisms is evident in the recovery process [25]. Therefore, a preliminary assessment should be conducted to determine whether the patient’s impaired functional parts are related to perception, memory, or more common semantic memory problems before implementing an intervention. Subsequent intervention should focus on the impaired functions of the patient. Currently, there are two strategies for intervention: compensatory strategies and remedial strategies (See Table 1). Compensatory strategies seek to intervene through facial memory and semantic memory, while remedial strategies target interventions through facial perception. It is important to note that although the concept of prosopagnosia has been recognized for many years, intervention approaches remain inconsistent and even more lacking for children. Therefore, generalizing interventions across different age groups still requires further evidence and validation in future research. It should be mentioned that the following strategies are proposed assuming prosopagnosia patients do not have co-occurring psychiatric conditions.

### 3.1. Compensatory Strategies

Compensatory strategies play a crucial role in assisting individuals with prosopagnosia to distinguish others by utilizing extra-facial information. These strategies can involve focusing on distinctive features, physical defects, specific body movements, voice, hairstyle, glasses, clothing, or ornamentation before encountering individuals [26]. Two research-backed strategies, namely Attention of Facial Features and Semantic Association, have been proven effective in this regard.

The Attention of Facial Features strategy focuses on guiding participants to direct their attention to the relevant facial regions they need to focus on. There are two evidence-supported approaches: Caricaturing and Feature Naming training. Caricaturing involves the presentation of exaggerated facial features during the face-learning phase. Computers can generate comic faces by exaggerating the difference between individual faces and ordinary faces [27,28]. This is based on a face space model [29,30]. Caricaturing reduces the similarity between the target face and other faces, resulting in faster and more accurate face recognition [31]. Mayer and Rossion utilized caricatured faces, unfamiliar adult faces, unfamiliar child faces, and familiar child faces to allow prosopagnosia patients to categorize and describe the characteristics of each face in each category [32]. After four months of training (twice a week), patients showed significant improvements in their ability to recognize faces using internal features. In another study, it was found that subjects trained with caricatured faces were more accurate in recognizing faces compared to those who were simply exposed to faces [33]. Recently, Limbach et al. combined perceptual training with specific parameters of deformed faces, which selectively exaggerated the shape or texture of faces [34]. This approach generated photo-realistic face pictures for subjects trained with the intervention and showed that parameter-specific caricatured face training improved the performance of individuals with poor face recognition and was transferable to untrained faces. Feature Naming training involves guiding participants during the learning phase through verbal instructions that include the name of the face and descriptions of its specific features. Face recognition relies on the holistic processing of the overall facial configuration. In contrast, part-based recognition approaches train patients to focus on local facial features rather than the whole configuration [35,36]. This can aid some prosopagnosia patients who have developed such strategies on their own, bypassing potentially impaired holistic face processing. This approach can compensate for the impairment of typical face recognition systems and may further facilitate a more natural adoption of compensatory strategies by patients. Brunsdon et al. used Feature Naming training to intervene with an eight-year-old patient diagnosed with prosopagnosia [37]. The patient was required to observe, discuss, and memorize five different features of each face, including age, gender, and three unique facial features. Following 14 training sessions over a one-month period, the patient’s recognition ability showed significant improvement, encompassing not only the initially trained faces but also images of faces viewed from various angles. Feature Naming training underscores the significance of focusing on facial features to enhance face recognition. Subsequently, a growing body of evidence supports the effectiveness of feature naming training in improving the ability to recognize familiar faces [33,38,39]. Overall, caricaturing modifies facial space and reduces face similarity to make faces more memorable, while Feature Naming training compensates for impaired configuration processing by directly highlighting facial features. These approaches hold the potential for benefiting individuals with prosopagnosia as well as those interested in enhancing their face recognition abilities. 

Semantic Association involves providing identity-related information, such as occupation and name, while participants are learning a face. The interactive activation model proposes that person identity nodes can access semantic memories specific to a person [40]. For patients, activating semantic information can facilitate face recognition even when the face itself fails to activate identification. The partial activation of semantic information can feedback to support the activation of the person identity node. Therefore, the purpose of this approach is to establish connections between personal identity nodes and specific semantic information, thus enhancing familiarity during the face recognition process. In simpler terms, the goal is to associate semantic information with each face to be remembered, which aids in improving recognition. Previous studies have demonstrated significant improvements in overt recognition among individuals with prosopagnosia when famous faces sharing identity-specific information, such as occupation, were presented [41]. Research has revealed that associating faces with person-related labels, such as names or occupations, leads to better face recognition performance than associating them with person-unrelated labels, such as object names or symbols. This underscores the importance of semantic information in elucidating our capacity to recognize familiar faces [42]. In summary, semantic association allows patients to compensate for impaired face recognition abilities by utilizing their intact semantic memory.

While compensatory strategies are beneficial for individuals with prosopagnosia, they may result in more laborious face recognition skills and might not easily generalize to untrained faces. Several factors, including the severity of symptoms, types of impairments, and individual differences such as age, can influence the effectiveness of these interventions [16]. Further research is necessary to determine the optimal conditions for employing compensatory strategies to improve face recognition and to identify ways to extend the application of these strategies to untrained faces.

### 3.2. Remedial Strategies

Remedial strategies, which aim to train more general skills that can be applied to any face, can facilitate more effective “normal” processing strategies and are typically used in patients with prosopagnosia due to impaired non-perceptual mechanisms that would be difficult to achieve otherwise [43]. These interventions can be divided into three strategies: Visual Scan Paths training, Face Feature Discrimination training, and Holistic Face training.

Visual Scan Paths training involves establishing correct face scanning paths by changing the eye movement trajectory of patients during face perception through games or tasks. Schwarzer et al. analyzed the eye movements of patients with prosopagnosia during face perception and found significant differences in the way they perceive faces compared to the general population [44]. Schmalzl et al. suggested that abnormalities in the visual scan paths for faces are likely to be a common feature in children with prosopagnosia [39]. A four-year-old and a six-year-old patient with DP received Visual Scan Paths training and demonstrated significant improvements in their ability to recognize target faces [45,46]. These findings suggest that establishing the correct visual scan paths for faces can enhance the ability to perceive internal facial features, particularly the eyes. The specific training for visual scan paths varies in different studies. Pizzamiglio et al. designed a task to search and clip eye images. Children were required to track different parts of each face (eyes, nose, mouth, facial contour, ears, and hair) in a specific order and quickly circle the eyes and clip out the eye area [46]. Di Vita et al. designed 16 explored intervention training programs presented in a game-like manner to help children. Two of these training programs also involved interventions in Visual Scan Paths training: The Eyes: “Search And Cut Out” and “Trace And Color The Faces”. The first training program also requires circling and cutting out the eye area of the face images, but only focuses on the eyes; the second training program requires participants to reproduce and color the facial features (eyes, nose, mouth, facial contour, ears) and hair of each face in a specific order [47]. However, they did not provide sufficient information about the efficiency of the interventions.

Internal Feature training entails focusing on the ability to discern various facial features, such as lip shape, eye color, or distances between facial features, such as the spacing between the eyes and eyebrows or the nose and mouth [48,49]. Internal facial features play an important role in face processing. In recent years, several studies have found that DP patients struggle to holistically integrate internal features and instead demonstrate more piecemeal feature sampling [50,51]. Research has also suggested that children’s reliance on internal features in faces may stem from a lack of facial experience [52]. This hints at the feasibility and potential of training focused on internal features. A study conducted distance discrimination training for facial features in 24 patients with DP and found that patients’ face perception was significantly improved [53]. There were four interventions specifically aimed at improving face feature recognition. The first intervention, called “Guess Who”, involved two patients selecting one of sixteen frontal photographs (eight females and eight males) and taking turns asking questions about the facial features in the picture to identify which image the other had chosen. The second intervention, “Spot the Differences”, required patients to identify differences between two altered face pictures, such as variations in eye color, lip thickness, and hairline. In the third intervention, “Same or Different”, children were presented with cards containing images of eyes, noses, and mouths, and were asked to identify whether there were differences between the cards and provide an explanation. The fourth intervention, “Face Puzzles”, required patients to combine images of a regular person’s face that had been divided into four, nine, or twelve parts. These training interventions helped patients process intrinsic facial features more effectively and had a greater impact on improving their face recognition ability [47,54].

Holistic Face training has also yielded positive outcomes, aiming to enhance the perceptual capacity of individuals with DP to discern the spatial arrangement of internal facial features across an expanded range [55]. Additionally, this training method has shown potential for improving the face recognition ability of children with AP. After receiving 14 weeks of online perception training, an eight-year-old girl found that her face perception skills were significantly improved. Eye movement analysis also showed that she spent more time observing internal facial features after the training [38]. The training also resulted in improved sensitivity of facial perception among participants, with some patients reporting lasting improvement in their daily lives for at least three months. However, the extent of improvement for untrained faces was comparatively less noticeable [56]. Further research is warranted to explore the long-term efficacy and generalizability of Holistic Face training across diverse populations.

The above studies indicated that remedial strategies targeting face perception can enhance face cognition in children with prosopagnosia, potentially extending these improvements to their everyday lives. However, it is important to acknowledge that remedial training may not benefit all children with prosopagnosia or improve all aspects of their face processing abilities. Nonetheless, these studies offer a promising direction for restoring the face processing system and provide hope for improving the quality of life for children with prosopagnosia. Future research should continue to investigate and refine these remedial strategies to optimize their effectiveness and applicability.

### 3.3. A New Approach: Wearable Devices for Prosopagnosia

With the rapid advancement of information technology, wearable devices have emerged as a significant innovation. Initially, research focused on developing wearable devices to assist visually impaired individuals in enhancing their social interactions [57,58,59]. These early devices aimed to alleviate the challenges faced by visually impaired individuals during social encounters. Over time, their application expanded to include brain-injured patients, such as those affected by stroke, Alzheimer’s disease, and other causes of prosopagnosia [60,61,62]. However, these wearable devices have primarily functioned as social aids, offering mechanical cues during face recognition, while neglecting the potential for rehabilitative capabilities in face recognition.

In response to this limitation, one researcher developed a new integrated wearable system to aid patients with prosopagnosia [63]. The system consists of an Android application that has both real-time face recognition and at-home training modes, and a wearable eyepiece that collects and transmits face information in real time through an external webcam. The real-time face recognition mode of the system detects the face of the person interacting with the patient and generates a unique face code that is compared with the contact face code list generated during pre-training. The at-home training mode allows patients to train and self-test using contact list face images in real-time face recognition mode. The training process includes feature naming training that combines memory and perception training and focuses on the internal features of patients. The study used the inverted face effect to simulate recognition difficulties of patients with prosopagnosia, and the results showed that training using the system can enhance subjects’ recognition of inverted face images, thus supporting the hypothesis that this training can improve face recognition ability. However, further research is necessary to determine the generalizability of these results to patients with prosopagnosia, particularly pediatric patients.

## 4. Future Directions

This paper presents a summary and comparison of potential interventions for children with prosopagnosia, which include compensatory strategies and remedial strategies. Compensatory strategies reviewed include Attention of Facial Features and Semantic Association, while remedial strategies reviewed include Visual Scan Path training, Internal Feature training, and Holistic Face training. Previous studies have shown that compensatory strategies may be more effective than remedial strategies for patients with AP, while both compensatory and remedial strategies showed some improvement in both children and adults with DP [16,53].

Despite showing some promise, the effectiveness of these interventions for children with prosopagnosia remains limited, and their applicability and efficacy are subject to various constraints. The lack of a widely accepted treatment for children with prosopagnosia emphasizes the need for further research to improve intervention strategies. This paper proposes three future research directions to enhance intervention methods for prosopagnosia. By focusing on these research directions, it is possible to significantly improve the effectiveness of interventions and enhance the quality of life of children with prosopagnosia. Nevertheless, additional research is required to fully understand the potential benefits of these approaches.

### 4.1. From Laboratory to Real Life

Overall, the current research and practice on interventions for children with prosopagnosia are still in their early stages, leaving room for further exploration and development. Compensatory and remedial strategies have shown potential benefits for children with prosopagnosia. However, before implementing interventions, it is crucial for researchers and practitioners to conduct thorough evaluations of the underlying cause of the child’s disorder, identify specific symptoms and areas of impairment, and accordingly tailor interventions. This individualized approach ensures that interventions address the unique needs and challenges of each child, maximizing their chances of improvement. 

In a study, children with prosopagnosia were trained for 18 months, but no improvement was observed in their face processing skills. The child in this study had impaired perceptual mechanisms, which made direct face perception training challenging [64]. Many current intervention methods are designed in laboratory settings and primarily rely on the recognition of face pictures to assess effectiveness. However, this approach is often sensitive to environmental factors and may not easily translate to daily life. Future interventions could consider using more ecologically valid materials, such as motion pictures and videos. While most current interventions require highly skilled professionals and are limited to small groups, the rise of the Internet has allowed some researchers to develop online interventions for prosopagnosia [65]. Future studies could explore combining online intervention systems with families and schools to improve intervention outcomes. 

### 4.2. Social Challenges for Children with Prosopagnosia

The inability to recognize and remember faces can result in significant impairments in social skills, causing patients to experience anxiety and depression [12]. Face recognition serves as the initial step for children to engage in face-to-face social interactions, and the impairment of this ability can significantly hinder their social development [66]. While current intervention studies have primarily focused on remedying damaged face recognition or memory skills, there has been comparatively less emphasis on addressing other factors that can negatively impact a child’s life, such as social interaction skills [12,13]. 

Research has shown that age and certain personality traits may be associated with reduced stress and anxiety levels caused by prosopagnosia [13]. Future investigations should include the study of age and personality factors in children with prosopagnosia, providing additional perspectives for addressing intervention issues related to anxiety, depression, and social relationships. In addition to current behavioral interventions, children with prosopagnosia also require supplementary methods to address their daily challenges, such as symptom disclosure to inform family, friends, classmates, and teachers about the condition, raising awareness of the effects of prosopagnosia on individuals. Future interventions may consider standardizing symptom disclosure methods and incorporating them into existing intervention programs, ensuring children’s safety, optimal education, and social interactions.

### 4.3. New Intervention Paths

Although current intervention methods for improving facial recognition abilities in children have shown effectiveness, they still have certain limitations. In the future, there is potential for the development of more diverse and efficient approaches to enhance children’s facial recognition abilities beyond simple behavioral interventions. Two promising methods that can supplement behavioral training have been identified: the intranasal administration of oxytocin and non-invasive brain stimulation [67,68].

Oxytocin is a neuropeptide that has been found to enhance social cognition by increasing the salience of social cues [69]. In one study, ten adult DP patients and ten matched control subjects were tested. Each participant underwent two testing sessions separated by 14–25 days. In each session, they inhaled either oxytocin or a placebo spray and completed two face-processing tests and the Multidimensional Mood Questionnaire (MMQ) to control for any mood effects. The results showed that patients improved in both face tests under the oxytocin condition [65]. It suggests that oxytocin may be a promising intervention method for improving face recognition in children with prosopagnosia.

Non-invasive brain stimulation comes in many forms, such as transcranial direct current stimulation (tDCS), transcranial random noise stimulation (tRNS), and galvanic vestibular stimulation (GVS), all of which have shown potential for intervention in prosopagnosia [16,70,71]. Among them, tDCS has been demonstrated to enhance performance in cognitive tasks [72]. Similarly, tRNS has been found to improve cognitive performance in motor and perceptual learning, and a combination of cognitive training with tRNS may enhance training effects [53,73]. Moreover, GVS also has some therapeutic effects. Research has shown that a 61-year-old patient with right-brain damage who was completely unable to recognize faces showed symptom relief in face perception after GVS treatment [74]. Moreover, some studies have provided theoretical rationale based on brain correlates for using non-invasive brain stimulation; e.g., N170, N250 [75]. The mechanisms behind these modalities likely involve altering cortical plasticity and excitability in regions critical for face processing. By selectively targeting key nodes in the distributed face processing network, non-invasive brain stimulation may rebalance neural circuits disrupted in prosopagnosia. Further research optimizing stimulation parameters and pairing with cognitive or perceptual training could unlock the full potential of these promising technologies for prosopagnosia intervention.

The intranasal inhalation of oxytocin and non-invasive brain stimulation can improve face recognition skills in the short term. However, neither tDCS nor tRNS has been widely used in the treatment of prosopagnosia. These techniques are still in their early stages and have only been applied to adult populations. As intervention methods continue to advance and mature, future research may consider combining these two methods with behavioral training and gradually introducing them into intervention training for children. Additionally, the potential mechanisms underlying the effects of oxytocin and non-invasive brain stimulation on face recognition should be investigated to optimize the intervention strategies. With continued advancements in technology and scientific understanding, the future holds promise for the development of more comprehensive and effective approaches to enhance facial recognition abilities in children with prosopagnosia.

## Figures and Tables

**Table 1:** Interventions for Children with Face Blindness (Prosopagnosia).

Intervention Type	Specific Strategies	Description
Compensatory Strategies	Attention of Facial Features	- Caricaturing: Exaggerating facial features during learning to increase distinction between faces - Feature naming: Verbal guidance to focus attention on specific facial features during learning
	Semantic Association	- Associating identity-related information (e.g., name, occupation) with faces during learning
Remedial Strategies	Visual Scan Paths Training	- Establishing correct face scanning patterns through games/tasks
	Face Feature Discrimination Training	- Improving the ability to discern facial features (e.g., eyes, nose, mouth)
	Holistic Face Training	- Enhancing the capacity to perceive the spatial arrangement of facial features

## Data Availability

Not applicable.
